# Extracellular Vesicles from the Probiotic Yeast *Pichia kudriavzevii*: Proteomic Characterization and Modulation of Immune and Defense Responses in an Induced Inflammation Model of Intestinal Epithelial Cells

**DOI:** 10.3390/nu18060912

**Published:** 2026-03-13

**Authors:** Angela Maione, Monica Matuozzo, Marianna Imparato, Chiara D’Ambrosio, Elisabetta de Alteriis, Marco Guida, Andrea Scaloni, Emilia Galdiero

**Affiliations:** 1 Department of Biology, University of Naples Federico II, 80126 Naples, Italy; angela.maione@unina.it (A.M.); marianna.imparato@unina.it (M.I.); dealteri@unina.it (E.d.A.); marco.guida@unina.it (M.G.); 2 Proteomics, Metabolomics and Mass Spectrometry Laboratory, Istituto per il Sistema Produzione Animale in Ambiente Mediterraneo (ISPAAM), National Research Council, 80055 Portici, Italy; monica.matuozzo@cnr.it (M.M.); andrea.scaloni@cnr.it (A.S.); 3 BAT Center-Interuniversity Center for Studies on Bioinspired Agro-Environmental Technology, University of Naples Federico II, 80055 Portici, Italy; 4 National Biodiversity Future Center (NBFC), 90133 Palermo, Italy

**Keywords:** probiotics, *Pichia kudriavzevii*, membrane vesicles, proteomics, immuno-inflammatory responses

## Abstract

**Background/Objectives**: Extracellular vesicles (EVs) derived from probiotics represent a new and exciting frontier in host-microbe therapeutics. These nanoscale carriers are not merely cellular byproducts but are sophisticated mediators of intercellular communication, capable of modulating immune responses, reducing inflammation, and inhibiting pathogens through a rich cargo of bioactive molecules. **Methods**: The EVs isolated from the culture supernatants of the yeast probiotic candidate *Pichia kudriavzevii* were characterized for their dimensions, protein composition, and targeting both the gut pathogen virulence and the host inflammatory response. **Results:** The vesicles had a size distribution from 100 to 150 nm, which is consistent with previous reports on fungal EVs. Proteomic analysis of the purified EVs identified a complex array of 189 proteins, hypothesized to be responsible for some of the antimicrobial and immunomodulatory properties observed. Safety was a key consideration, and the isolated EVs demonstrated no cytotoxicity in human Caco-2 cells and no in vivo toxicity in the *Galleria mellonella* larval model, confirming their potential for safe use. **Conclusions**: The field is moving towards a new era of “postbiotics,” where cell-free therapies offer a safer, more stable alternative to live probiotics.

## 1. Introduction

Extracellular vesicles (EVs) are nano-sized particles released by almost all cell types that are bordered by a phospholipid bilayer and contain lipids, proteins, carbohydrates, nucleic acids, pigments, toxins and other small molecules [1]. Initially identified as exosomes, the term “EVs” now broadly includes exosomes, ectosomes, and apoptotic bodies, reflecting their diverse origin, size, and molecular content. EVs act as key mediators of intercellular communication, transporting lipids, proteins, nucleic acids, and metabolites either locally or to distant districts. Due to their biological versatility, EVs have significant potential in the pharmaceutical industry, regenerative medicine, and cancer diagnostics. More recently, they have gained attention in the food sector for their ability to deliver compounds in a targeted manner. While most EVs studied so far are derived from animal fluids, fermented foods are emerging as a novel source of EVs, potentially playing a crucial role in microbe-host interactions [2,3,4,5,6,7,8,9].

Probiotics are living microorganisms that provide beneficial effects to the host, usually by mediating changes in the gut microbiota. Over the past few decades, their popularity and consumption have significantly increased due to their reported health benefits [10]. Moreover, probiotics were shown to positively influence interactions within the gut–brain axis [11,12]. Changes in microbiota composition were associated with various diseases, including inflammatory bowel disease (IBD), obesity, and irritable bowel syndrome (IBS) [13]. Therefore, the involvement of an altered microbiota in disease progression has sparked strong interest in exploring the therapeutic potential of probiotic strains. Recent studies have demonstrated that the administration of specific probiotic species can induce beneficial responses in models of colitis, IBS, and colorectal tumorigenesis, as the primary effect of probiotics is to modulate the host immune system, thereby preventing and treating infections [14,15].

Despite the benefits and general safety of probiotics, the consumption of living bacteria may cause adverse events, particularly in patients with pre-existing medical conditions or weakened immune systems. In such individuals, probiotic therapy may worsen inflammatory responses and convert probiotic bacteria into harmful microorganisms [16,17]. In this context, the latest microbiota-based therapies include the use of postbiotics, non-viable bacterial components and products derived from beneficial microbes that confer health benefits to the host [18,19]. This group includes cell fragments, biomolecules, and secreted bioactive compounds.

In this research field, EVs released by probiotic strains are emerging as potential new postbiotics. Recent research has expanded to explore the role of probiotic EVs in a wider range of functions by influencing the surrounding gut microbiota [20,21,22,23,24,25]. Some studies have provided evidence for the role of microbiota-derived EVs as key players in interkingdom communication within the gut, acting as mediators of microbiota functions on the host [26]. Specifically, EVs produced by probiotic bacteria, such as lactic acid bacteria (LAB), have been shown to exert beneficial immunomodulatory properties, as demonstrated in studies using inflammatory colitis models and intestinal immune cells [6]. Moreover, LAB-EVs showed numerous advantageous attributes, including cell-free characteristics, minimal toxicity, exceptional biocompatibility, low immunogenicity, non-replicative nature, and precise cellular targeting capabilities [27,28].

Similar studies on EVs produced by probiotic yeasts have been rarely accomplished. In general, most investigations focused on vesicles from pathogenic yeasts, such as *Cryptococcus neoformans* and *Candida albicans*, showing that these microorganisms, after infecting the human body, secrete EVs necessary for effective host colonization [29,30]. Since the latter studies have indicated that microbial EVs can interact with human cells, it is plausible to hypothesize that vesicles from probiotic yeasts might also contribute to the microorganism bioactivity.

In our previous studies, we isolated from home-made kefir grains a strain of *Pichia kudriavzevii* that was investigated for its probiotic characteristics. This yeast strain demonstrated tolerance to gastric acids and bile salts, indicating its ability to survive the stomach environment, reach the intestine, and potentially colonize this organ thanks to its auto-aggregation and co-aggregation properties. Furthermore, it was able to adhere in vitro to intestinal cell lines Caco-2 and HT-29, and it also showed biofilm-forming ability. The strain safety was confirmed in vitro, showing no haemolytic activity, resistance to common antibiotics, and sensitivity to antifungal agents. Its safety was further validated in vivo using *Galleria mellonella* larvae. By means of intestinal cell models, we also observed that both the live yeast and its cell-free supernatant inhibit the adhesion and invasion of a pathogenic *Salmonella* strain in Caco-2 cells. Additionally, they were able to modulate the production of pro- and anti-inflammatory cytokines in cells stimulated with Salmonella lipopolysaccharide (LPS), as well as regulate the expression of proteins involved in maintaining the intestinal barrier integrity [31,32].

In this study, we isolated EVs from the cell-free supernatant of the above-reported *P. kudriavzevii* probiotic strain to evaluate whether its previously reported anti-Salmonella and immunomodulatory properties might be associated with these vesicles. Therefore, we focused on: (i) the purification and biophysical characterization of *P. kudriavzevii* EVs; (ii) the evaluation of their proteinaceous cargo; (iii) the verification of their possible adverse effects on the host in selected in vitro and in vivo models; (iv) the assessment of the inhibition of both adhesion and invasion of Salmonella into Caco-2 cell lines; (v) the investigation on the triggered immune response by determining the secretion of pro-inflammatory and anti-inflammatory cytokines. Through this comprehensive study, we aimed to provide original insights into the biological role of EVs in *P. kudriavzevii*, eventually supporting their potential use as safe and effective next-generation postbiotics.

## 2. Materials and Methods

### 2.1. Strains and Culture Conditions

*P. kudriavzevii* strain CBS 5147 was isolated from homemade kefir samples as previously reported [31]. It was maintained in YPD agar (1% *w*/*v* yeast extract, 2% *w*/*v* bacto peptone (Gibco, Life Technologies Co., Detroit, MI, USA), 2% *w*/*v* glucose and 2% *w*/*v* agar (Sigma Aldrich, Co., St. Louis, MO, USA); a single colony was used to inoculate YPD broth at 37 °C under shaking, overnight. The culture was then centrifuged, washed three times with sterile phosphate-buffered saline (PBS), and cells were resuspended in non-supplemented Dulbecco’s Modified Eagle’s Medium (DMEM; Sigma-Aldrich, St. Louis, MO, USA) at different concentrations for the next experiments. *Salmonella enterica* serovar Derby, obtained from a food isolate [33], was kept in our laboratory Culture Collection and routinely grown in tryptone soy broth (TSB; Oxoid, Basingstoke, UK) at 37 °C for 18 h.

### 2.2. EVs Isolation and Characterization

EVs were isolated from the supernatants of *P. kudriavzevii* cultures grown in YPD medium. Initially, culture supernatants were cleared of cells and debris by centrifugation at 8000× *g* for 20 min, at 4 °C. The resulting cell-free supernatants were then concentrated using Amicon ultrafiltration devices with a 100 kDa molecular weight cut-off (Merck, Darmstadt, Germany), followed by filtration through Ultrafree-CL centrifugal filters with a pore size of 0.45 µm (Sigma-Aldrich). To verify the complete removal of fungal cells, aliquots of the filtered supernatants were plated onto YPD agar and incubated to monitor for yeast growth. The concentrated and filtered supernatants were subsequently subjected to ultracentrifugation at 100,000× *g* for 1 h, at 4 °C. The resulting pellets were washed once in sterile PBS by repeating the ultracentrifugation step under the same conditions. Final EV pellets were resuspended in 100 µL of sterile PBS. All ultracentrifugation steps were performed using an Optima MAX-XP ultracentrifuge (Beckman Coulter, Brea, CA, USA) equipped with a TLA 100.3 rotor (Beckman Coulter, Brea, CA, USA). The isolated EVs were stored at −80 °C until further analysis.

EVs characterization was performed using Nanoparticle Tracking Analysis (NTA). EV size distribution and concentration were measured with the Nanosight NS300 platform (Malvern Panalytical, Malvern, UK) equipped with a sCMOS camera (Andor Technology, Belfast, UK) and a green laser, operating under the control of NTA software v. 3.4. Samples were diluted 1:100 in PBS and analyzed with a camera level set to 16 and a detection threshold of 4.

Dynamic light scattering (DLS) was used to measure the zeta potential of EVs with a Zetasizer Nano-ZS (Malvern Instruments, Worcestershire, UK). Samples were prepared at a concentration of 1.12 ± 0.35 × 10^9^ particles/mL. Measurements were carried out at 25 °C using a 4 mW He–Ne laser (633 nm) with a fixed scattering angle of 173°. Videos were recorded to visualize EVs in each sample.

### 2.3. Protein Extraction, Quantification and Fractionation

For protein extraction, *P. kudriavzevii* EVs (2 biological samples) were lysed in parallel with 8 M urea, 1% *w*/*v* SDS, containing complete EDTA-free protease inhibitor cocktail (Roche, Mannheim, Germany), at a 1:10 *v*/*v* ratio. EVs’ lysis was initially performed by probe sonication (0.3 cycles) on ice for 2 min, followed by a bath sonication on ice for an additional 2 min, to enhance lysis efficiency. To ensure complete lysis, the latter step was repeated twice, and finally, the sample was left on ice for 30 min. Supernatants were collected by centrifugation at 6000× *g* for 15 min, at 4 °C, and the corresponding protein concentration was determined with the Pierce BCA Protein assay kit™ (Thermo Fisher Scientific, San Jose, CA, USA), according to the manufacturer’s instructions. Finally, proteins were precipitated with 6 vol of cold acetone overnight at −20 °C. After precipitation, proteins were pelleted by centrifugation at 8000× *g* for 20 min, at 4 °C, and then vacuum-dried with a SpeedVac roto-evaporator (Thermo Fisher Scientific). Recovered proteins were resolved with preparative 12% T SDS-PAGE under reducing conditions. After protein staining with colloidal Coomassie blue and destaining, whole gel lanes (two in number) were cut into 15 slices, which were separately triturated, washed with water and acetonitrile, treated with 55 mM iodoacetamide in 100 mM NH_4_HCO_3_, washed again, and finally digested with trypsin (12.5 ng/µL) in 50 mM NH_4_HCO_3_. Resulting peptide mixtures were desalted/concentrated using ZipTip μC18 (Millipore, Burlington, MA, USA), vacuum-dried and finally reconstituted in 5% *v*/*v* formic acid.

### 2.4. Mass Spectrometry

Peptide mixtures were analyzed by means of a nanoUltraHighPressure liquid chromatography (nUHPLC)-electrospray (ESI)-Q-Orbitrap-MS/MS platform consisting of a Vanquish-Neo nano-chromatographer (Thermo Fisher Scientific) linked to an Exploris 480 mass spectrometer (Thermo Fisher Scientific) through an easy-spray ion source. Peptides were loaded on an EASY-Spray C18 column (250 mm × 75 μm ID, 2 μm particles, 100 Å pore size) (Thermo Fisher Scientific), and eluted with a gradient of solvent B (19.92/80/0.08 *v*/*v*/*v* water/acetonitrile/formic acid) in solvent A (99.9/0.1 *v*/*v* water/formic acid), at a flow rate of 250 nL/min. The gradient of solvent B started at 6%, increased to 31% over 90 min, increased to 50% over 5 min, increased to 95% over 5 min, remained at 95% for 8 min, and finally returned to 6% for the equilibrating step. The mass spectrometer operated in data-dependent acquisition mode collecting MS spectra of positive ions within a scan *m*/*z* range of 375–1200, using a nominal resolution of 120,000 full width at half maximum (FWHM), an automatic gain control (AGC) target of 3 × 10^6^ ions, a maximum injection time (IT) of 50 ms, and a dynamic exclusion value of 45 s, which was followed by MS/MS scans of the 20 most abundant ions. MS/MS spectra were acquired using a normalized collision energy (HCD) of 30%, a resolution of 15,000 FWHM, an AGC target of 2 × 10^5^ ions, and a maximum IT of 100 ms.

### 2.5. Bioinformatics

For protein identification, raw mass data files were merged into Proteome Discoverer v. 3.1 software (Thermo Fisher Scientific, San Jose, CA, USA), enabling database search with Mascot algorithm v. 2.4.2 (Matrix Science, London, UK). Database searching was performed with the following parameters: *P. kudriavzevii* UniProtKB sequence database (17,479 protein entries), including the most common protein contaminants; carbamidomethylation at Cys as fixed modification; oxidation at Met, deamidation at Asn and Gln, and pyroglutamate formation at Gln as variable modifications. Peptide mass tolerance and fragment mass tolerance were set to ±10 ppm and ±0.02 Da, respectively. Trypsin was set as the proteolytic enzyme, and the maximum number of missed cleavages was limited to 2. Protein candidates assigned based on at least one sequenced peptide, 2 PSMs and a Mascot score ≥ 25 were considered confidently identified. Definitive peptide assignment was always associated with manual spectra visualization and verification. Results were filtered to 1% false discovery rate.

Proteomic data were deposited in the ProteomeXchange Consortium via the PRIDE partner repository [34] with the dataset identifier PXD069498.

Identified *P. kudriavzevii* vesicle proteins were associated with components of EVs already assigned in other organisms through a dedicated search in the ExVe database [35]. STRING software v. 12.0 was used to investigate and visualize the functional relationships among all identified *P. kudriavzevii* vesicle proteins [36]. Functional enrichment analysis, subcellular localization assignment, and further information on biological pathways/molecular processes regarding identified *P. kudriavzevii* EV proteins were achieved by using dedicated tools assigning diverse Gene Ontology (GO) categories [37], and COMPARTMENTS [38] and Reactome [39] resources available within the STRING software.

### 2.6. Human Intestinal Cell Lines and Stimulation Conditions

Human intestinal epithelial cells, Caco-2, purchased from the American Type Culture Collection (ATCC, Manassas, VA, USA), were cultured in DMEM containing 10% fetal bovine serum (FBS), 2 mM L-glutamine, and 1% penicillin–streptomycin (Sigma Aldrich Co., St. Louis, MO, USA), and maintained at 37 °C, in a 5% CO_2_ atmosphere. Caco-2 cells were seeded on 96-well plates at a concentration of 1 × 10^3^ per well and grew to 80% of confluence for all experiments. Lipopolysaccharide (LPS) (Sigma Aldrich, St. Louis, MO, USA) of *S. typhimurium* (10 μg/mL) was used to stimulate Caco-2 cells to detect reactive oxygen species (ROS) and to determine the release of nitric oxide (NO) and cytokines.

### 2.7. Evaluation of EVs In Vitro and In Vivo Safety

The in vitro cytotoxicity of *P. kudriavzevii* EVs was evaluated on Caco-2 cells using the MTT assay. Cells were seeded at a density of 2 × 10^4^ cells/well in 96-well plates and incubated overnight to allow adhesion. EVs were added at concentrations of 10^5^, 10^6^, 10^7^, 10^8^ and 10^9^ EVs/mL. After the exposure period, MTT reagent (Sigma Aldrich, St. Louis, MO, USA) was added and incubated for 3–4 h. The resulting formazan crystals were dissolved, and absorbance was measured at 570 nm using a microplate reader (Varioskan LUX Multimode Microplate Reader Thermo Fisher Scientific USA). Cell viability percentages were calculated relative to untreated control cells.

The in vivo safety of *P. kudriavzevii* EVs was assessed using *Galleria mellonella* larvae. For each experimental group, ten randomly selected larvae at a comparable developmental stage were used. A Hamilton syringe was used to inject 10 µL of sample—containing between 10^5^ and 10^9^ yeast EVs, suspended in sterile PBS—into the left hind proleg of each larva. Larval survival was monitored at 37 °C over a 3-day period. The experiment was conducted in three independent biological replicates.

### 2.8. Adhesion and Invasiveness Assays

The ability of *P. kudriavzevii* EVs to modulate the adhesion and invasiveness of *S*. Derby was evaluated using Caco-2 cells seeded at a density of 2.5 × 10^5^ cells/mL in antibiotic-free DMEM, according to our previous study [31,32]. After 24 h of incubation to allow cell monolayer formation, cells were pre-treated with EVs at a concentration range from 10^5^ to 10^9^ EVs/mL for 2 h, at 37 °C, in a 5% CO_2_ atmosphere. Subsequently, *S.* Derby was added at a multiplicity of infection (MOI) of 1:100 and incubated for an additional 2 h under the same conditions. At the end of this period, non-adherent bacteria were removed by washing the monolayers three times with sterile PBS. To evaluate adhesion, the cells were detached with trypsin, and the resulting samples were serially diluted and plated on Salmonella Chromogenic Agar Base supplemented with Salmonella Selective Supplement (Oxoid, Basingstoke, UK). Plates were incubated at 37 °C overnight, and colony-forming units (CFUs/mL) were counted to quantify the total cell-associated bacteria.

To assess bacterial invasion, after the initial infection phase and PBS washes, the monolayers were incubated for an additional 2 h in DMEM supplemented with 250 µg/mL gentamicin sulfate (Sigma-Aldrich) to eliminate extracellular bacteria. The cells were then lysed with 100 µL of Triton X-100 and processed as described above to quantify the internalized Salmonella (CFUs/mL).

### 2.9. Lactate Dehydrogenase (LDH) Assay

To evaluate cell membrane damage in Caco-2 cells induced by *S*. Derby infection, with or without 10^8^ EVs/mL pre-treatment, LDH release was measured under the same infection conditions described above. After treatments, culture supernatants were collected and LDH levels were quantified using a colorimetric assay kit (Sigma Aldrich, Co., St. Louis, MO, USA), following the manufacturer’s instructions. Absorbance was read at 490 nm using a microplate reader (Varioskan LUX Multimode Microplate Reader, Thermo Fisher Scientific USA).

### 2.10. NO Production, Intracellular and Mitochondrial ROS Detection in Caco-2 Cells

The ability of *P. kudriavzevii* EVs to modulate NO production in Caco-2 cells was assessed using the Griess assay. Cells (2 × 10^3^ cells per well) were seeded into 96-well microtiter plates. The following day, the culture medium was removed and replaced with fresh medium containing either (i) a mixture of EVs (10^8^ EVs/mL) and 10 µg/mL of *S. typhimurium* LPS (pre-incubation), (ii) EVs alone (10^8^ EVs/mL) or (iii) LPS alone. After 24 h of incubation at 37 °C and under 5% CO_2_ atmosphere, cell supernatants were collected. Nitrite concentrations were determined via a colorimetric reaction using the Griess Reagent Kit for nitrite quantification (Invitrogen™, Frederick, MD, USA). Absorbance was measured at 548 nm using a Varioskan LUX Multimode Microplate Reader (Thermo Fisher Scientific, San Jose, CA, USA).

Intracellular and mitochondrial ROS levels were evaluated using the fluorescent probes DCFH-DA and MitoSOX Red, respectively [40]. Cells were cultured and treated as described above. Following the manufacturer’s instructions, cells were harvested, washed twice with PBS, and incubated with either 2′,7′-dichlorofluorescein diacetate (DCFH-DA, 10 μM in PBS; Molecular Probes, Eugene, OR, USA) for 30 min, at 37 °C, in the dark, or with MitoSOX Red (5 μM in HBSS; Invitrogen, Thermo Fisher Scientific, Waltham, MA, USA) for 20 min, at 37 °C, in the dark. After three washes with PBS, fluorescence intensity was measured using a microplate reader: for DCFH-DA, excitation/emission was set at 488 nm/540 nm; for MitoSOX Red, excitation/emission was set at 510/580 nm. Results were expressed as fold change in fluorescence intensity relative to untreated control cells.

### 2.11. Quantification of Cytokines by ELISA

To evaluate the anti-inflammatory properties of *P. kudriavzevii* EVs on cytokine levels in LPS-treated cells, Caco2 cells were seeded in a 12-well plate and incubated for 24 h. The following day, the cells were treated with LPS (10 µg/mL), EVs alone (10^8^/mL), or cells pre-treated with EVs and then stimulated with LPS. Untreated cells served as the control group. After 24 h, culture supernatants were collected for the quantification of cytokine levels. ELISA kits (Elabscience, Wuhan Elabscience Biotechnology Co., Wuhan, China) were used to measure the concentrations of IL-6 (Cat. n: E-EL-H6156), IL-4 (Cat n: E-EL-H0101), IL-10 (Cat n: E-EL-H6154), IL-8 (Cat n: E-EL-H6008), and IL-1β (Cat n: E-EL-H0149), following the manufacturer’s instructions. Absorbance was measured at 490 nm using a microplate spectrophotometer. Cytokine concentrations in the test samples were evaluated with reference to standard curves.

### 2.12. Statistical Analysis

Statistical analyses were performed using GraphPad Prism software (version 8.02 for Windows; GraphPad Software, La Jolla, CA, USA; www.graphpad.com). Results are expressed as mean ± standard deviation (SD) of at least three independent experiments. Survival curves were analyzed using the Kaplan–Meier method. Comparisons between groups were performed using one-way ANOVA followed by Dunnett’s post hoc test. A *p*-value of less than 0.05 was considered statistically significant.

## 3. Results

### 3.1. Structural Characterization and Quantification of EVs from the Foodborne Probiotic Yeast P. kudriavzevii

**Figure 1:**
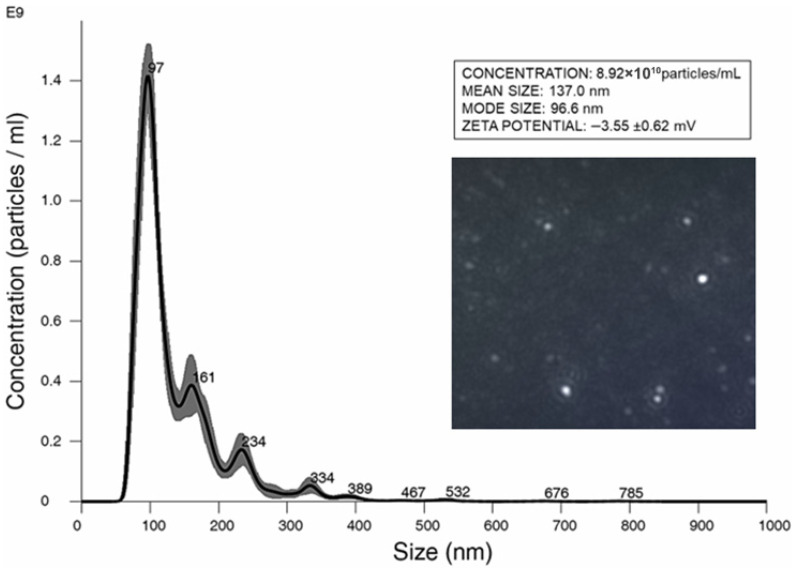
Size distribution and concentration of *P. kudriavzevii* EVs, as determined by Nanoparticle Tracking Analysis (Nanosight NS300, Malvern Panalytical, UK). The zeta potential of EVs was measured by Dynamic Light Scattering (DLS) using a Zetasizer Nano-ZS (Malvern Instruments, Worcestershire, UK).

An increasing number of studies suggest that microorganisms can influence human cells by secreting EVs [4]. This report explores the possible occurrence of this phenomenon for *P. kudriavzevii* EVs, which were first isolated from yeast liquid cultures, confirming that vesicle preparations did not contain any viable cells. Their size distribution and concentration were determined using Nanoparticle Tracking Analysis (NTA) (Figure 1). The analysis showed that the *P. kudriavzevii* strain produced EVs with a mean diameter of 137.3 ± 4.5 nm. Based on the EV concentration in the samples, as also measured by NTA, the efficiency of vesicle isolation was estimated to be 8.92 ± 2.83 × 10^10^ particles/mL. The measured zeta potential value was −3.55 ± 0.62 mV.

### 3.2. Proteomic Analysis of EVs from the Probiotic Yeast P. kudriavzevii

A proteomic investigation was accomplished to characterize the protein cargo of *P. kudriavzevii* EVs. Briefly, vesicle proteins were extracted, separated by SDS-PAGE, *in-gel* digested with trypsin, and the resulting peptides were finally analyzed by nUHPLC-ESI-Q-Orbitrap-MS/MS. Bioinformatics of the resulting MS data allowed the identification of a total of 189 *P. kudriavzevii* proteins. A dedicated search against the ExVe database [35] recognized several of them (136 in number, corresponding to 72% of the whole assigned molecules) as components of the protein cargo of EVs from other microorganisms.

**Figure 2:**
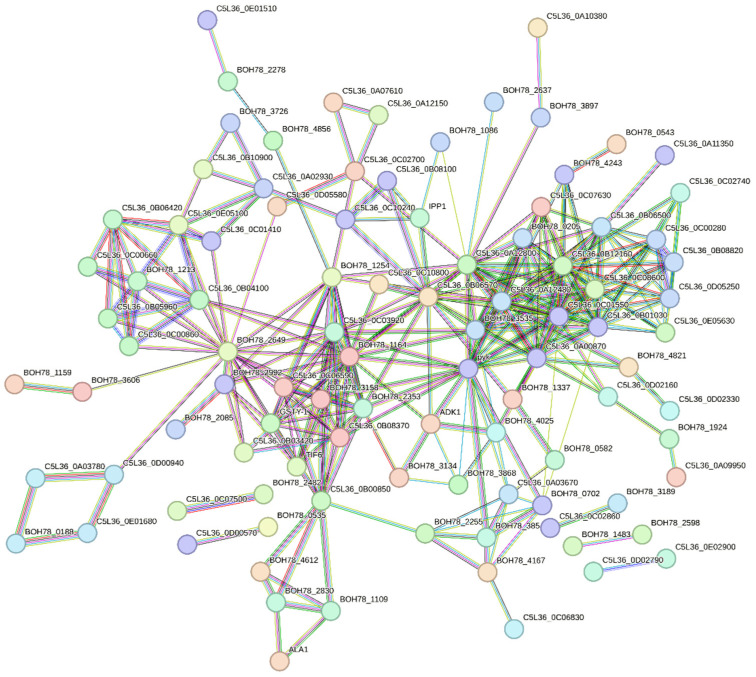
Protein–protein interaction (PPI) network of proteins identified in *P. kudriavzevii* EVs using STRING software. The network shows functional protein associations assigned with a high-confidence threshold (0.700). BOH78_3158: ribosomal_L18e/L15P domain-containing protein; BOH78_1164: 40S ribosomal protein S3; C5L36_0B08370: 40S ribosomal protein S4; BOH78_3606: 5-methyltetrahydropteroyltriglutamate-homocysteine methyltransferase; C5L36_0C07630: 6-phosphogluconate dehydrogenase, decarboxylating; C5L36_0C06590: 60S ribosomal protein L13; C5L36_0A09950: acetyl-CoA hydrolase; BOH78_1337: aconitate hydratase, mitochondrial; C5L36_0C02700: uncharacterized protein; BOH78_3134: adenosine kinase; BOH78_1159: adenosyl homocysteinase; ADK1: adenylate kinase; C5L36_0A07610: uncharacterized protein; C5L36_0D05580: ADP-ribosylation factor; ALA1: alanine-tRNA ligase; BOH78_0543: alcohol dehydrogenase 4, mitochondrial; BOH78_4612: asparagine-tRNA ligase, cytoplasmic; BOH78_4167: aspartate aminotransferase; BOH78_4821: aspartate-semialdehyde dehydrogenase; C5L36_0B06570: ATP synthase subunit alpha; C5L36_0C10800: uncharacterized protein; C5L36_0A10380: uncharacterized protein; BOH78_0535: cerevisin; BOH78_1254: elongation factor 1-alpha; BOH78_2649: elongation factor 3; C5L36_0E05100: uncharacterized protein; C5L36_0B10900: uncharacterized protein; C5L36_0B03420: eukaryotic translation initiation factor 3 subunit A; TIF6: eukaryotic translation initiation factor 6; C5L36_0A12150: F-actin-capping protein subunit beta; C5L36_0C07500: uncharacterized protein; BOH78_2482: 3-hydroxyacyl-[acyl-carrier-protein] dehydratase; C5L36_0C08600: fructose-bisphosphate aldolase; BOH78_1483: glucan 1,3-beta-glucosidase; BOH78_2598: glucan 1,3-beta-glucosidase; C5L36_0E05630: glucose-6-phosphate 1-epimerase; C5L36_0B12160: glucose-6-phosphate isomerase; BOH78_2255: glutamate dehydrogenase; C5L36_0B00850: GST C-terminal domain-containing protein; GSTY-1: glutathione S-transferase; C5L36_0A12800: glyceraldehyde-3-phosphate dehydrogenase; BOH78_3868: Golgi apyrase; BOH78_4856: GTP-binding nuclear protein; C5L36_0B05960: heat shock protein 70 1; C5L36_0C00860: heat shock protein SSA2; C5L36_0B04100: heat shock protein SSB1; C5L36_0C00660: uncharacterized protein; BOH78_1213: heat shock protein SSE1; C5L36_0B06420: hsp82-like protein; BOH78_2278: histone H2A; BOH78_1924: homocitrate synthase, cytosolic isozyme; IPP1: inorganic pyrophosphatase; BOH78_0582: isocitrate lyase; BOH78_1109: isoleucine-tRNA ligase, cytoplasmic; C5L36_0C03920: 60S acidic ribosomal protein P0; BOH78_2353: ribosomal_L2_C domain-containing protein; BOH78_2830: leucine-tRNA ligase, cytoplasmic; C5L36_0E02900: lysophospholipase; C5L36_0D02790: lysophospholipase; BOH78_3854: malate dehydrogenase; C5L36_0C02740: mannose-6-phosphate isomerase; C5L36_0D02160: glutamine amidotransferase type-1 domain-containing protein; BOH78_4025: nucleoside diphosphate kinase; C5L36_0D02330: ornithine aminotransferase; C5L36_0C06830: M20_dimer domain-containing protein; C5L36_0A03780: peptidyl-prolyl cis-trans isomerase; BOH78_0188: peptidylprolyl isomerase; C5L36_0D00940: peptidylprolyl isomerase; C5L36_0E01680: PPIase cyclophilin-type domain-containing protein; BOH78_3189: peroxiredoxin TSA1; C5L36_0A03670: uncharacterized protein; C5L36_0B06500: uncharacterized protein; C5L36_0A12480: phosphoglycerate kinase; BOH78_0205: phosphoglycerate mutase; BOH78_3535: enolase 1; BOH78_2637: phosphoserine phosphatase; C5L36_0B08820: phosphotransferase; C5L36_0C00280: phosphotransferase; C5L36_0D05250: phosphotransferase; BOH78_1086: pasma membrane ATPase; BOH78_2085: polyadenylate-binding protein; BOH78_3897: protein BMH; BOH78_3726: protein disulfide-isomerase domain; C5L36_0A02930: protein disulfide-isomerase; BOH78_0702: pyruvate carboxylase; BOH78_4243: pyruvate decarboxylase isozyme 3; pyk: pyruvate kinase; BOH78_2992: ATP-dependent RNA helicase eIF4A; C5L36_0E01510: uncharacterized protein; C5L36_0C02860: superoxide dismutase; C5L36_0B01030: transaldolase; C5L36_0C01550: transketolase; C5L36_0A00870: triosephosphate isomerase; C5L36_0A11350: uncharacterized protein; C5L36_0C01410: uncharacterized protein; C5L36_0B08100: uncharacterized protein; C5L36_0C10240: vacuolar proton pump subunit B; C5L36_0D00570: saccharopepsin.

To investigate the functional relationships among all identified *P. kudriavzevii* vesicle proteins, a protein–protein interaction (PPI) network was generated using the STRING software and searching the corresponding organism database [36]. A high-confidence interaction score threshold (0.700) was applied to include a broad but reliable set of functional associations. The resulting output included a highly connected central network that comprised 88 proteins, suggesting that many vesicular components are functionally linked in coordinated biological processes (Figure 2). An additional five disconnected binary networks were also detected. This network was found to be statistically significant, with a PPI enrichment *p*-value < 1 × 10^−16^ indicating that the observed degree of connectivity was far greater than expected.

**Figure 3:**
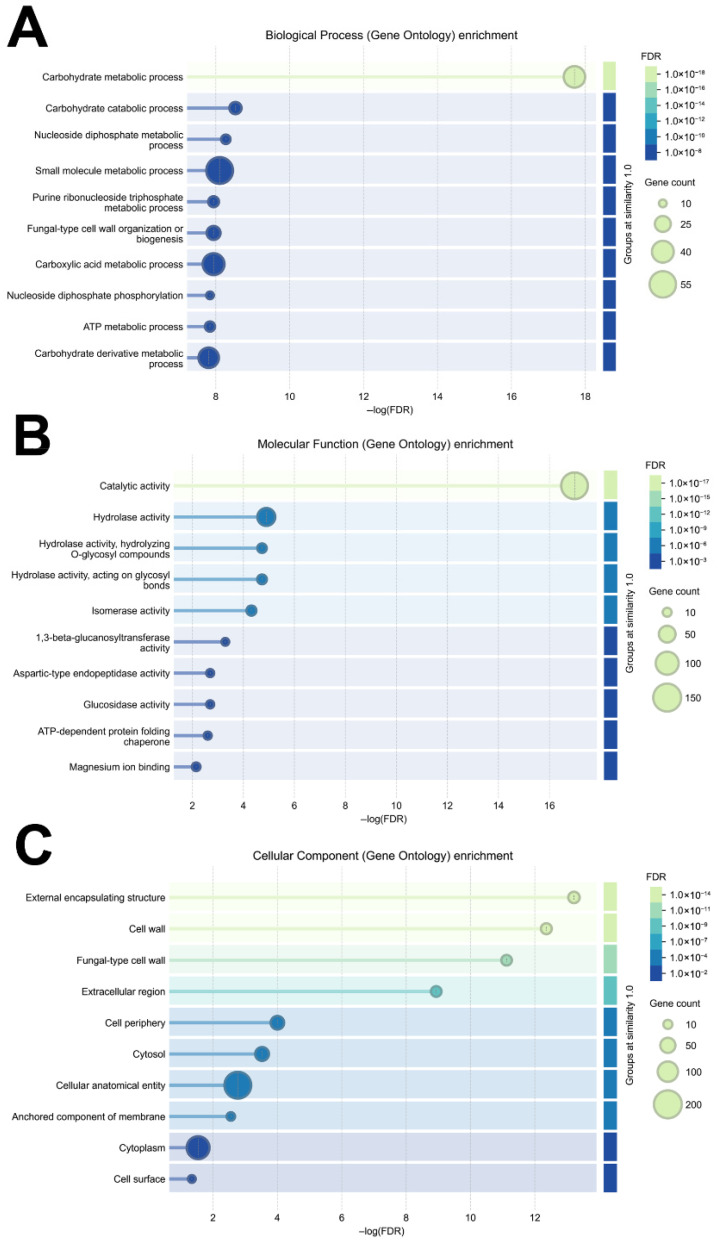
Enriched Biological Process (BP) (panel (**A**)), Molecular Function (MF) (panel (**B**)) and Cellular Component (CC) (panel (**C**)) Gene Ontology terms associated with proteins identified in *P. kudriavzevii* EVs. The figure shows the most significantly enriched BP, MF and CC terms based on false discovery rate (FDR) (color scale) and number of associated genes (bubble dimension).

Furthermore, a functional enrichment analysis was carried out on *P. kudriavzevii* vesicle proteins, focusing on Biological Process (BP), Molecular Function (MF), and Cellular Component (CC) Gene Ontology (GO) categories [37] (Figure 3). Regarding the BP-GO category, the analysis highlighted a significant enrichment in biological processes that, according to the false discovery rate values and the number of associated proteins, were in order related to carbohydrate metabolism, nucleotide/nucleotide metabolism, small molecule metabolism and fungal-type cell wall organization/biogenesis (Figure 3A). On the other hand, a functional enrichment for the MF-GO category revealed a significant representation of catalytic activities, confirming the predominant role of enzymes within the *P. kudriavzevii* EV protein cargo (Figure 3B). Among the enriched enzymatic functions worth mentioning is the hydrolase activity and its subcategories related to the hydrolysis of O-glycosyl compounds and glycosidic bonds. Other relevant enzymatic activities included the isomerase, 1,3-beta-glucanosyltransferase, glucosidase, and aspartic-type endopeptidase ones. Altogether, these data indicated that *P. kudriavzevii* vesicle proteins are strongly oriented toward catalytic activities capable of modulating the structure of polysaccharides and glycoproteins. Finally, functional enrichment analysis for the CC-GO category revealed a significant representation of extracellular structures and components associated with the fungal cell wall, as in the case of “external encapsulating structure”, “cell wall”, “fungal-type cell wall”, “extracellular region”, “cell periphery” and “anchored component of membrane” terms (Figure 3C). Further enrichments in the CC-GO category were also observed for “cytosol” and “cytoplasm” terms, although these were represented at more modest levels.

**Figure 4:**
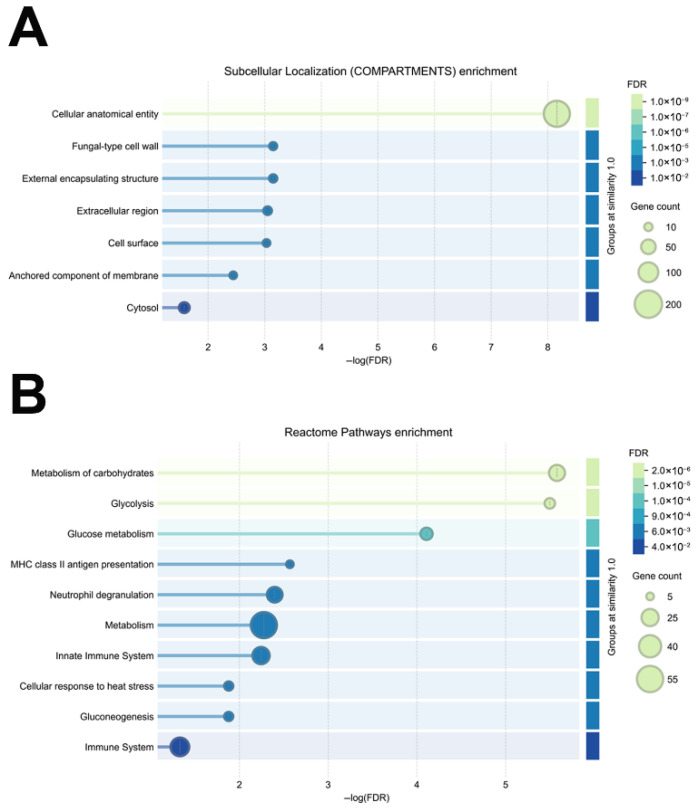
Subcellular localization (**A**) and Reactome (**B**) enrichment analysis of proteins identified in *P. kudriavzevii* EVs. The figure shows the most significantly enriched terms based on false discovery rate (FDR) (color scale) and number of associated genes (bubble dimension).

The latter results were further confirmed by subcellular localization enrichment analysis using the COMPARTMENTS resource [38], which highlighted a significant representation of proteins associated with fungal-type cell wall structures and extracellular encapsulating regions. In fact, the terms “fungal-type cell wall”, “external encapsulating structure”, “extracellular region” and “cell surface” displayed significant FDR values. Enrichment of the above-reported terms and of the “anchored component of membrane” one suggested a possible involvement of *P. kudriavzevii* EVs in extracellular protein trafficking and intercellular communication processes (Figure 4A).

A functional enrichment analysis was also carried out using the Reactome resource, a manually curated database for biological pathways and molecular processes. Although primarily focused on human proteins, Reactome was also used for non-human species according to structural/functional homology criteria [39], enabling the identification of enriched biological pathways in various organisms. This specific analysis revealed that *P. kudriavzevii* EV proteins are enriched in terms related to energy metabolism (“metabolism of carbohydrates”, “glycolysis”, “glucose metabolism”, “metabolism” and “gluconeogenesis”), immune activation response (“MHC class II antigen presentation”, “neutrophil degranulation”, “innate immune system” and “immune system”), and cell defense processes (Figure 4B). These results confirmed the above-reported functional and localization enrichment analyses overall indicating a significant representation of proteins involved in metabolic pathways modulating the structure of sugars, polysaccharides and glycoproteins in *P. kudriavzevii* EVs, which might occur at fungal-type cell wall and extracellular regions, and could potentially modulate immune response, cell adhesion and stress signaling processes.

### 3.3. Evaluation of P. kudriavzevii EVs In Vivo and In Vitro Toxicity

**Figure 5:**
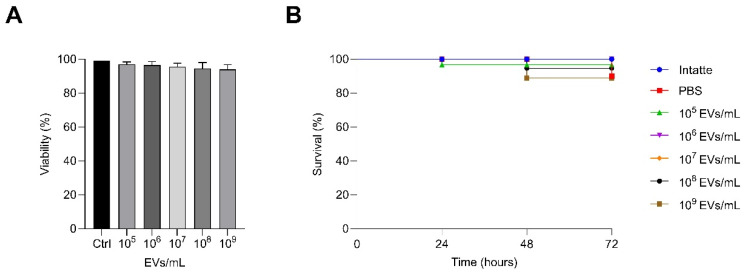
(**A**) Cytotoxicity effect of *P. kudriavzevii* EVs at concentrations of 10^5^, 10^6^, 10^7^, 10^8^ and 10^9^ particles/mL on Caco-2 cells as measured with the MTT assay. (**B**) Cytotoxicity in *Galleria mellonella* larvae injected with EVs at concentrations ranging from 10^5^ to 10^9^ per larva.

Since assessing the composition of EVs is not sufficient on its own to derive information on their bioactivity, we evaluated vesicle effects on the host and determined the potential toxicity of these particles to host cells. To this end, both an in vivo system employing the model organism *G. mellonella* and an in vitro model using human epithelial cells were used. The impact of *P. kudriavzevii* EVs on the survival of *G. mellonella* larvae was monitored for three days following injection of EVs at concentrations ranging from 10^5^ to 10^9^ per larva; injections consisted of proper EV amounts dissolved in 10 µL of PBS. In the case of 10^6^, 10^7^, and 10^8^ EV concentrations, no statistically significant differences in larval survival were observed when compared to the control group. A slight, but statistically non-significant, reduction in survival was noted only in the group injected with the highest concentration (10^9^ EVs) (Figure 5A). Furthermore, the potential cytotoxic effect of *P. kudriavzevii* EVs on the human epithelial cell line Caco-2 was assessed using the MTT assay (Figure 5B). Incubation with various EV concentrations led to a slight decrease in metabolic activity, although the effect was not statistically significant.

### 3.4. P. kudriavzevii EVs Inhibit Some In Vitro Virulence Traits of Salmonella

**Figure 6:**
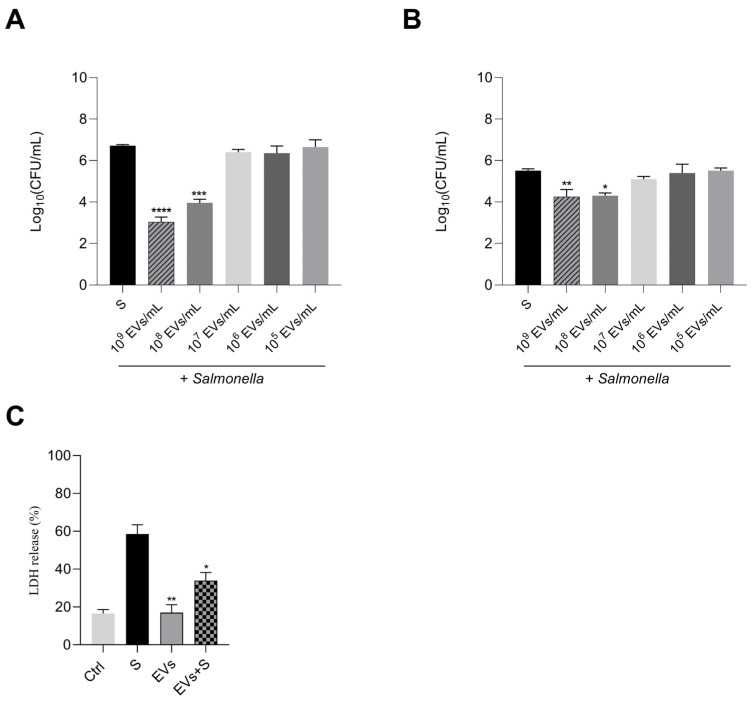
In vitro Salmonella adhesion and invasion studies. (**A**) Effect of different concentrations of *P. kudriavzevii* EVs (10^9^, 10^8^, 10^7^, 10^6^ and 10^5^ particles/mL) on the adhesion of *S*. Derby. to Caco-2 cells. The data presented are the means of three independent assays with three wells per assay. (**B**) Invasion of Salmonella on Caco-2 cells pre-treated with different concentrations of *P. kudriavzevii* EVs (10^9^, 10^8^, 10^7^, 10^6^ and 10^5^ particles/mL). (**C**) EVs concentration at 10^8^ particles/mL significantly inhibited the release of LDH in Caco-2 cells. The 100% LDH value corresponds to the maximal LDH release obtained from Caco-2 cells completely lysed with Triton X-100; Ctrl: Caco-2 cells without treatment; S: cells infected with Salmonella; EVs: cells treated with EVs; EVs + S: cells pre-treated with EVs and then infected with Salmonella. The data presented are the means of three independent assays with three wells per assay. Asterisks indicate significant difference vs. cells infected with Salmonella (* = *p* < 0.05, ** = *p* < 0.01, *** = *p* < 0.001, **** = *p* < 0.0001, Dunnet’s test).

The ability to colonize host cells is one of the main characteristics of Salmonella. Therefore, we tested a foodborne Salmonella strain to evaluate its capacity to colonize Caco-2 cells, either pre-treated or not with *P. kudriavzevii* EVs. As shown in Figure 6A, *Salmonella* exhibited an adhesion capacity of approximately 6.7 log_10_ (CFU/mL) under control conditions; this value significantly decreased when EVs were used at concentrations of 10^9^ or 10^8^ mL^−1^, dropping to about 3 log_10_ (CFU/mL) and about 4 log_10_ (CFU/mL), respectively. At lower EV concentrations, the decrease in adhesion capacity was not significant and remained similar across these conditions.

The ability to inhibit Salmonella invasion in Caco-2 cells pre-treated with different concentrations of *P. kudriavzevii* EVs was also evaluated. As shown in Figure 6B, a significant reduction in invasiveness consistently occurred at the concentrations of 10^9^ (~4.5 log_10_ CFUs/mL) or 10^8^ (~4 log_10_ CFUs/mL), compared to the control. The pre-treatment had no significant effect at lower concentrations of 10^7^, 10^6^ or 10^5^ EVs mL^−1^.

The protective properties of *P. kudriavzevii* EVs on Caco-2 cells were also confirmed by measuring the corresponding LDH release. Caco-2 cells were first pre-treated with yeast EVs and subsequently infected with Salmonella. Results from these experiments indicate that cell infection with Salmonella alone caused LDH release of 58.5 ± 4.9% more than control. However, pre-exposure of Caco-2 cells to yeast EVs at concentrations of 10^8^ mL^−1^ significantly reduced foodborne bacterial cytotoxicity since LDH release was below 40% (Figure 6C).

Altogether, these data clearly show that *P. kudriavzevii* EVs presented a potential for preventing *Salmonella* interaction with intestinal cells, whether this bacterium was inadvertently ingested with food.

### 3.5. P. kudriavzevii EVs Inhibit LPS-Induced Generation of Intracellular/Mitochondrial ROS

**Figure 7:**
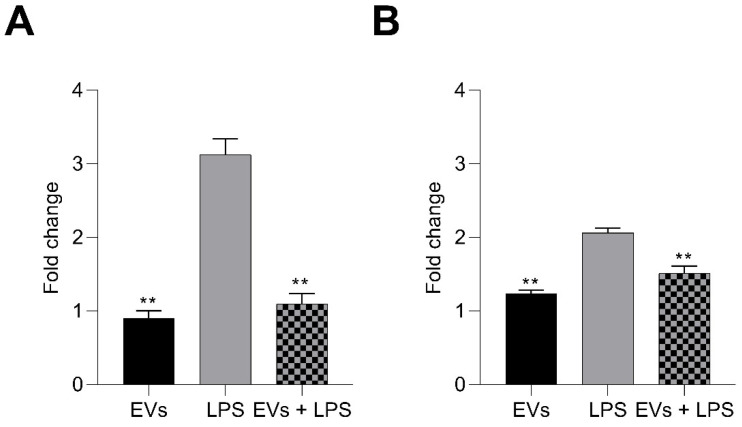
Effects of *P. kudriavzevii* EVs on LPS-induced ROS generation. Intracellular (**A**) and mitochondrial (**B**) ROS levels in Caco-2 cells were detected using DCFH-DA and MitoSOX Red staining, respectively. Data refer to cells stimulated with LPS or pre-treated with EVs and then stimulated with LPS; data for cells treated with EVs alone are also reported. Results are expressed as fold change with respect to the control. ** = *p* < 0.01 (Dunnett’s test).

To investigate the potential effects of *P. kudriavzevii* EVs on the generation of oxidant compounds in LPS-stimulated Caco-2 cells, DCFH-DA and MitoSOX Red staining were used to assess the corresponding intracellular and mitochondrial ROS levels, respectively. As shown in Figure 7, the mean fluorescence intensities of DCF (panel A) and MitoSOX (panel B) were dramatically increased upon stimulation with the LPS, compared to the control group. However, pre-treatment with yeast EVs at a concentration of 10^8^ mL^−1^ effectively reversed these effects.

### 3.6. Effects of P. kudriavzevii EVs on NO Production

**Figure 8:**
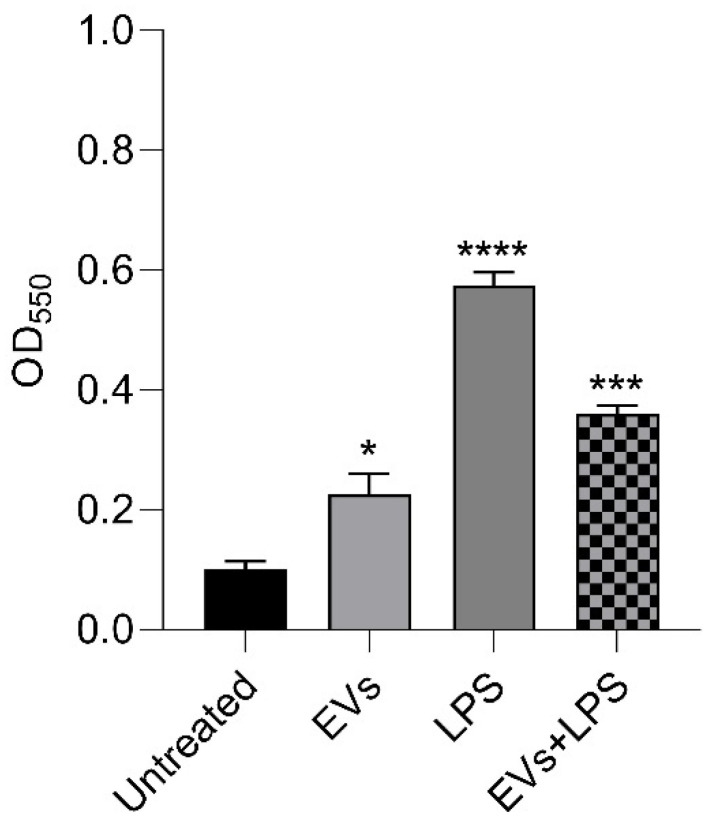
Effects of EVs on the release of pro-inflammatory mediator NO in Caco-2 cells. The data presented are the mean values of three independent experiments. * *p* < 0.05, *** *p* < 0.001, **** *p* < 0.0001 (Dunnet’s test).

To assess the potential anti-inflammatory capacity of *P. kudriavzevii* EVs, the effect of yeast vesicles on nitrous oxide (NO) production in LPS-induced Caco-2 cells was examined. Caco-2 cells were treated with LPS alone, EVs alone, or a combination of EVs and LPS. For NO detection in the experimental samples, nitrite production was measured in the supernatants using the Griess method, evaluating compound concentration after incubation with a red-violet azo dye. As shown in Figure 8, LPS treatment determined a significant increase in NO production in Caco-2 cells compared to the control. The pre-incubation of cells with *P. kudriavzevii* EVs significantly reduced NO production compared to cells treated with LPS alone. Finally, a minimal increase in nitrous oxide production was observed when Caco-2 cells were treated with yeast EVs alone.

### 3.7. Effect of P. kudriavzevii EVs on IL-1β, IL-4, IL-6, IL-8 and IL-10 Levels

**Figure 9:**
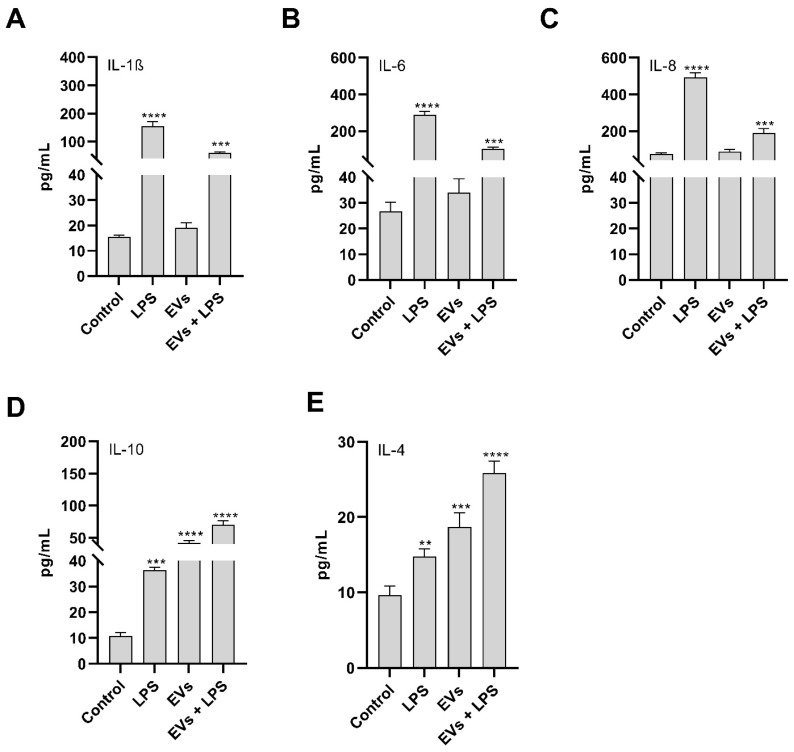
In vitro evaluation of the immunomodulatory activity of *P. kudriavzevii* EVs. Shown are the levels of: (**A**) IL-1β, (**B**) IL-6, (**C**) IL-8, (**D**) IL-10, and (**E**) IL-4 in untreated cells (control), cells stimulated with LPS (LPS), cells treated with EVs alone (EVs) or cells pre-treated with EVs after stimulation with LPS (EVs + LPS). ** = *p* < 0.01, *** = *p* < 0.001, **** = *p* < 0.0001 (Dunnet’s test).

To investigate the response of Caco-2 cells to *P. kudriavzevii* EVs under inflammatory conditions, the human cell line was treated with LPS to mimic an inflammatory environment. Accordingly, LPS treatment determined a significant concentration increase in some pro-inflammatory cytokines (Figure 9), which was consistently and significantly reduced when cells were pre-treated with LPS in the co-presence of EVs at a concentration of 10^8^ particles/mL. More specifically, while LPS stimulation induced oxidative stress leading to a significantly increased production of the pro-inflammatory cytokine IL-8, pre-treatment of cells with EVs resulted in a marked inhibition of IL-8 release, thereby demonstrating the anti-inflammatory potential of *P. kudriavzevii* EVs (Figure 9C). These findings indicate that EVs may modulate the innate immune response at the intestinal epithelium by attenuating oxidative stress-induced cytokine expression. A similar trend was also observed for measured IL-1β and IL-6 concentrations in the culture supernatants as detected via ELISA. LPS treatment alone significantly elevated cytokine levels compared to the untreated control, whereas cells pre-treated with yeast EVs exhibited a significant reduction in IL-1β and IL-6 concentrations, with respect to LPS-stimulated cells (Figure 9A,B). Taken together, these results strongly supported the anti-inflammatory activity of *P. kudriavzevii* EVs.

Given that enteric bacteria activate IL-10-producing cells and that high IL-10 levels suppress pathological immune responses and promote gut immune tolerance, we further assessed the ability of yeast EVs to modulate IL-10 secretion in LPS-stimulated Caco-2 cells. As illustrated in Figure 9D, IL-10 secretion increased in LPS-treated cells, compared to the untreated control. However, pre-treatment with EVs prior to LPS stimulation significantly increased IL-10 levels, further confirming the anti-inflammatory properties of *P. kudriavzevii* EVs. A similar effect was also observed for the anti-inflammatory cytokine IL-4, which resulted in increased cells following pre-treatment with EVs (Figure 9E).

## 4. Discussion

The presence of EVs in the culture supernatants of bacteria and fungi has been studied in increasing detail in recent years; in the case of pathogenic organisms, these vesicles have been directly related to the release of molecules necessary for virulence and/or associated with immune modulation [41,42,43]. In parallel, recent scientific advances have revealed many mechanisms by which probiotics exert health-promoting effects in humans and laboratory animals [44]. Among these mechanisms, probiotic-derived EVs have emerged as crucial mediators of the host–microbe communication. Rich in proteins, nucleic acids, and lipids, these nanoscale vesicles carry bioactive molecules capable of influencing gene expression, metabolic activity, and disease outcomes in the host [45].

Current research on probiotic EVs focuses on their molecular composition, regulatory mechanisms, and therapeutic potential. Studies have demonstrated their roles in intercellular communication, drug delivery, immune modulation, inflammation reduction, and the management of intestinal disorders [6,46,47,48,49,50,51]. Moreover, they hold promise in cancer therapy and vaccine development [52]. Although still in early stages, ongoing research is expected to deepen our understanding of probiotic EVs and support their future integration into clinical applications. Nowadays, the literature indicates that the biological effects of probiotic EVs closely resemble those of the probiotics themselves, suggesting a potential role for EVs in mediating probiotic mechanisms [53]. Notably, proteins contained within these vesicles can bind to host cell receptors, triggering intracellular signaling pathways involved in immune regulation, cell proliferation, and apoptosis [6]. Additionally, these proteins may interact with ligands on target cells, thereby modulating cellular metabolism and gene expression [27].

EVs from LAB probiotics have been widely studied so far [20,21,23,25,28,54]. In some cases, these investigations showed that EVs contain proteins that could mediate pathogen inhibition, and in this way, may possibly compete with pathogens for colonization in the intestine. On the other hand, comparative proteomic analyses of EVs from different LABs demonstrated that the protein composition of EVs can be very different among species [55]. Interestingly, antimicrobial bacteriocins were detected in EVs from *L. acidophilus* ATCC 53544 [56]. Moreover, a study on *Lacticaseibacillus casei* BL23-derived EVs identified various functional proteins, including enzymes, signaling molecules, heat shock proteins and adhesion-related proteins that may significantly influence host cellular processes [57,58,59]. Differently, the study and characterization of EVs from probiotic yeasts is still in its infancy.

In this work, we have hypothesized that EVs from the isolated probiotic yeast *P. kudriavzevii* should contain cargo molecules possibly involved in antimicrobial and immunomodulatory properties targeting an antibiotic-resistant enteric pathogen, such as Salmonella. On this basis, we characterized dimensions, protein composition and immunomodulatory activity of the purified EVs. In addition, we demonstrated that pre-treatment with *P. kudriavzevii* EVs reduces the virulence of Salmonella in a cell model of infection. The size distribution of EVs observed in the present study (100–150 nm) aligns with previous reports on fungal EVs [60]. Further, we have here demonstrated that the isolated EVs do not exhibit cytotoxicity in human Caco-2 cells, nor do they show in vivo toxicity, as assessed using the *G. mellonella* larval model. In vitro pre-treatment of Caco-2 cells with high concentrations of EVs led to a reduction in the adhesion capacity of a foodborne pathogenic Salmonella strain. Furthermore, the same pre-treated cells exhibited not only a decreased level of bacterial invasion, but also a lower release of LDH, indicating a reduced cell damage following infection. The above-reported results are parallel with those from studies on EVs from LAB, whereas they are original in the case of vesicles from yeast probiotics. As far as the active EV concentration is concerned, our results demonstrate that already at 10^8^ particles/mL, we found a significant reduction in adhesion and invasion of Salmonella together with a relevant immunomodulatory effect. Therefore, this concentration can be taken into consideration in the possible formulation of *P. kudriavzevii* EV-based postbiotic.

Analysis of redox stress, both at the total and mitochondrial level, revealed a marked decrease in ROS concentration in EV-pre-treated cells compared to those stimulated with LPS, with values approaching those of untreated control cells. Similarly, NO production was significantly reduced in EVpre-treated cells compared to both control and LPS-stimulated cells. Pre-treatment with EVs in LPS-stimulated cells resulted in a decreased expression of the pro-inflammatory cytokines IL-6, IL-8, and IL-1β, alongside an increase in the anti-inflammatory cytokines IL-10 and IL-4. These findings support the immunomodulatory potential of EVs derived from a *P. kudriavzevii* strain, also suggesting that several pathways could be involved in the EVs–cell interaction.

Our results reveal a complex array of protein components in EVs, which might modulate these activities. Although proteomic studies on EVs from pathogenic fungi, such as *Cryptococcus deuterogattii* and *Histoplasma capsulatum*, have revealed the presence of proteins involved in immune modulation, stress response, and metabolism [61,62], corresponding data for food-grade or probiotic yeasts remain scarce. Addressing this knowledge gap was essential to validate the role of *P. kudriavzevii* EVs as functional postbiotics and support their potential applications in gut health and immunomodulation.

Analysis of the protein cargo of *P. kudriavzevii* EVs uncovered a broad array of functionally relevant proteins. A total of 189 proteins were identified, many of which are involved in key biological functions, such as metabolic pathways modulating the structure of sugars, polysaccharides and glycoproteins, which might occur at fungal-type cell wall and extracellular regions, and could potentially modulate immune response, cell adhesion and stress signaling processes. These proteins reflect the metabolic activity of fungal EVs and suggest a potential role in host-microbe and microbe-microbe interactions in the gut environment. The detection of glycolytic enzymes within the protein cargo of EVs is a recurring observation across various species, including yeasts [63,64] and bacteria [65]. Furthermore, studies on human exosomes have demonstrated their ability to synthesize ATP via glycolysis, suggesting that this energy production may play a key role in the uptake of extracellular vesicles by recipient cells [66]. Consistent with these findings, some glycolytic enzymes have been identified in EVs from most yeast species examined to date [63], a result corroborated in the present work for *P. kudriavzevii*. Further, Mierzejewska and co-authors reported that glycolytic enzymes such as enolase and glyceraldehyde-3-phosphate dehydrogenase (GAPDH), commonly found in EVs from pathogenic fungi such as *Candida albicans*, were also detected in vesicles from the probiotic *S. boulardii* [67]. In the present study, both proteins have also been identified in *P. kudriavzevii* EVs. Their presence might also be attributed to the reported moonlighting nature of them, having different functions according to their cellular location [68,69]. Thus, GAPDH has already been reported to be involved in adhesion functions and enolase participating in multiple cellular processes, such as vacuolar membrane fusion, beyond its primary metabolic function [70,71].

The proteomic analysis of the cargo of *P. kudriavzevii* EVs also showed a significant representation of extracellular structures and components associated with the fungal cell wall, similarly to what was observed for vesicles from the probiotic *S. boulardii* [72] and from potential probiotic strains of *S. cerevisiae* isolated from fermented foods [73]. Examining the composition of *P. kudriavzevii* EVs, the identification of hsp70, hspSSA2, hspSSB1, hspSSE1 and hsp82-like supports the role of such heat shock proteins in fungal adaptation, as these chaperones are known to mediate stress responses, protein folding, and antifungal resistance [74,75]. Vesicular traffic in eukaryotes is a complex and multifunctional cellular mechanism essential for intracellular transport [76]. Intracellular vesicles facilitate the movement of proteins destined for secretion, moving them along pathways that originate at the endoplasmic reticulum and proceed to the Golgi complex. From the Golgi, proteins are sorted via the *trans*-Golgi network for delivery to the cell surface. The precise and regulated secretion of such diverse molecules, which can include various enzymes and signaling proteins, significantly impacts the interaction of fungal cells with their hosts. As an example, and in confirmation of the literature, Golgi apyrase here found in *P. kudriavzevii* EVs plays a crucial role in controlling this intricate vesicular transport system. On the other hand, elongation factor 1-alpha was frequently found in the proteomes of fungal EVs and those of other microorganisms. Its presence in *P. kudriavzevii* EVs may reflect the cytoplasmic origin of the vesicles, but it might also be associated with a potential regulatory role in cell-to-cell communication, adhesion, or host immune response, as already suggested in previous studies [77,78,79,80].

Although research describing the interaction between bacterial ribosomal proteins (RPs) and host cells is still in its early stages, parallels have been observed in eukaryotic systems, where RPs act as host cell receptors and thereby modulate microbial pathogenesis [81]. Given that recent studies have shown that several proteins present in EVs are likely involved in immunomodulatory effects, including RPs, our study suggests that, under stress conditions, *P. kudriavzevii* ribosomal proteins are actively secreted and perform moonlighting functions. In particular, they may contribute to the stabilization of biofilm structure by binding to extracellular DNA and act as decoys to neutralize antibiotics, thereby enhancing drug tolerance within the microbial community, as already observed [82,83,84]. Moreover, some RPs themselves were shown to function as antimicrobial peptides [85]. This dual role suggests that *P. kudriavzevii* RPs not only provide collective defense against exogenous therapeutic agents, but may also act as secreted vesicular toxins, potentially conferring a competitive advantage by inhibiting or eliminating other microbial competitors in the highly competitive extracellular environment [85].

## 5. Conclusions

In conclusion, this study provides new insights into the biological properties and potential therapeutic relevance of extracellular vesicles derived from the probiotic yeast *Pichia kudriavzevii*. Further investigation into the unidentified proteins will be necessary in future research, as well as other studies employing optimized isolation and functional assays will be crucial to clarify the role of the EV cargo in the interaction with the host. Moreover, the therapeutic efficacy of *P. kudriavzevii* EVs on intestinal cells in vitro requires additional validation. The therapeutic efficacy of *P. kudriazevii* EVs requires additional study not only in vitro but also in vivo, to test their potential clinical applications. Considering their proven efficacy against Salmonella, it would be of interest to validate the EVs preparation against other pathogenic *Enterobacteriaceae* and in the management of gastrointestinal disorders, for which proper models are available. From the perspective of aproper formulation (e.g., in oral capsules), the stability in the gastrointestinal conditions will also need to be analyzed.

However, despite these limitations, taken together, our results reveal that EVs from this probiotic yeast are not only inert vesicles but rather dynamic entities carrying bioactive proteins with potential roles in metabolism, host interaction, and immune regulation, and support their potential use as next-generation postbiotics for therapeutic application in gut-related diseases.

## Data Availability

The data presented in this study are available on request from the corresponding author due to ongoing research and unpublished data.
